# Case Report: "ADHD Trainer": the mobile application that enhances cognitive skills in ADHD patients

**DOI:** 10.12688/f1000research.5689.3

**Published:** 2015-06-23

**Authors:** Gonzalo Ruiz-Manrique, Kazuhiro Tajima-Pozo, Francisco Montañes-Rada

**Affiliations:** 1 Department of Psychiatry, Hospital Universitario Fundacion Alcorcon, Alcorcon, 28922, Spain

**Keywords:** ADHD; mobile app; TCT method; working memory

## Abstract

We report the case of a 10 year old patient diagnosed with attention deficit hyperactivity disorder (ADHD) and comorbid video game addiction, who was treated with medication combined with a novel cognitive training method based on video games called TCT method. A great risk of developing video game or internet addiction has been reported in children, especially in children with ADHD. Despite this risk, we hypothesize that the good use of these new technologies might be useful to develop new methods of cognitive training. The cognitive areas in which a greater improvement was observed through the use of video games were visuospatial working memory and fine motor skills. TCT method is a cognitive training method that enhances cognitive skills such as attention, working memory, processing speed, calculation ability, reasoning, and visuomotor coordination. The purpose of reviewing this case is to highlight that regular cognitive computerized training in ADHD patients may improve some of their cognitive symptoms and might be helpful for treating video game addiction.

## Introduction

Attention deficit hyperactivity disorder (ADHD) is the most commonly diagnosed neurodevelopmental disorder in childhood, which affects 3% to 7% of the population worldwide ^1^. ADHD is characterized by distractibility, hyperactivity and impulsivity. The standard treatment for ADHD includes mainly medication, psychosocial and behavioral treatment, and cognitive training exercises.

Cognitive training exercises are especially useful when cognitive impairment is observed and when a regular and personalized cognitive training is performed ^2^. Studies in participants with cognitive impairment have shown that regular and daily cognitive training can improve some of their cognitive symptoms ^3, 4^. In addition, recent studies have demonstrated that computerized working memory and executive function training programs lead to better results than ordinary cognitive training methods in children with ADHD ^5– 7^.

Children’s use of electronic devices, Internet and video games, has noticeably increased in the last 10 years. Since the first case of Internet addiction was described in 1996 by Young ^6^, several other pathologies have been proposed including pathological gambling and dependence ^7^. Despite extensive research literature available, the prevalence and proper diagnostic criteria for pathological gaming are still debated among the scientific community ^8^. Gaming addiction represents part of the postulated construct of Internet addiction, and is the most widely studied specific form of Internet addiction to date ^9^. Prevalence estimates range from 2% ^10^ to 15% ^11^, depending on the respective socio-cultural context, sample, and assessment criteria utilized. A great risk of developing video game or Internet addiction has been reported in children, and especially in those with ADHD ^8^. Stimulants such as methylphenidate (MPH), given to treat ADHD, and video game play have been found reduce Internet use in subjects with co-occurring ADHD and Internet video game addictions ^9^.

Despite the risk of Internet addiction we hypothesize that good use of these new technologies can be useful to develop new methods of cognitive training useful in to treat ADHD an Internet addiction.

## Case report

This case study involves a 10 year old child born in Madrid (Spain) who received treatment in a childhood psychiatry unit for 2 years due to behavioral disorders and ADHD. No other previous medical history was reported. His mother, aged 35, received psychological treatment for anxiety 3 years ago. His father, aged 36, works as an engineer and presented no relevant medical history. The patient was their only son. The parents described a great addiction to video games in the last year, referring 4 hours per day of video game playing, affecting his social interaction, and causing a lack of imaginative play and poor academic scores. Teachers at the school reported deterioration in his academic performance over the past year. At that time, the child was treated with methylphenidate 40 mg per day. The patient’s parents reported to the psychiatrist that the only significant change from the previous year was a major addiction to a war videogame.

To reduce the exposure to video games, we used a novel technique, based on the Tajima Cognitive Method (TCT) called “ADHD Trainer”. It consists in a cognitive stimulation program with a mobile/tablet application designed specifically to treat ADHD.

Behavioral and academic improvements were rated on the Conners Parent and Teacher Rating Scales (brief version) and Barkley School Situations Questionnaire.

ADHD diagnosis was made according to DSM V criteria ^10, 11^. Attention was rated with CPT Conners Conners Continuous Performance Test.

Differential diagnosis between oppositional defiant disorder and ADHD disorder was considered, because most of the symptoms were observed at home, however not angry or irritable mood was observed.

The patient was treated with a combination of methylphenidate and cognitive training method based in the TCT method. The patient received daily treatment with 40 mg of methylphenidate, and at least 10 minutes of daily cognitive training with the “ADHD Trainer” app.

**Figure 1.**
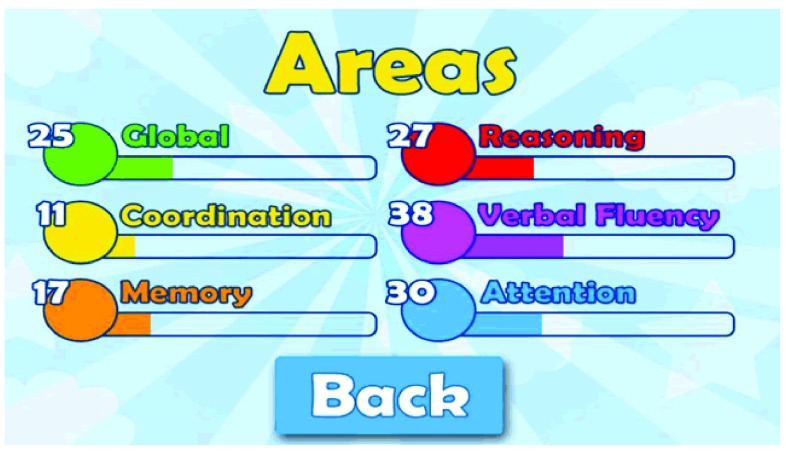
The cognitive areas treated with *ADHD* Trainer.

The TCT is a type of computer adaptive test (CAT), as it adapts to the individual’s cognitive strengths and weaknesses, based on his own scores over time, as well as those of his peers. Users receive separate scores in different cognitive areas, including simple calculation, attention, perceptual reasoning, and visuomotor coordination ( Figure 1). The goal of the daily training is to reach a pre-set individualized score in different cognitive domains, in order to complete a week of successful training. The exercises comprising “ADHD Trainer” are described in the following Table 1.

**Table 1.** Exercises comprising "ADHD Trainer from Monday to Sunday. 10 minutes per day.

Monday	Tuesday	Wednesday	Thursday	Friday	Saturday	Sunday
Attention Memory	Calculation Visuo motor coordination Perceptual reasoning	Attention Memory	Calculation Visuo motor coordination Perceptual reasoning	Attention Memory	Calculation Visuo motor coordination Perceptual reasoning	Attention Memory

**Figure 2.**
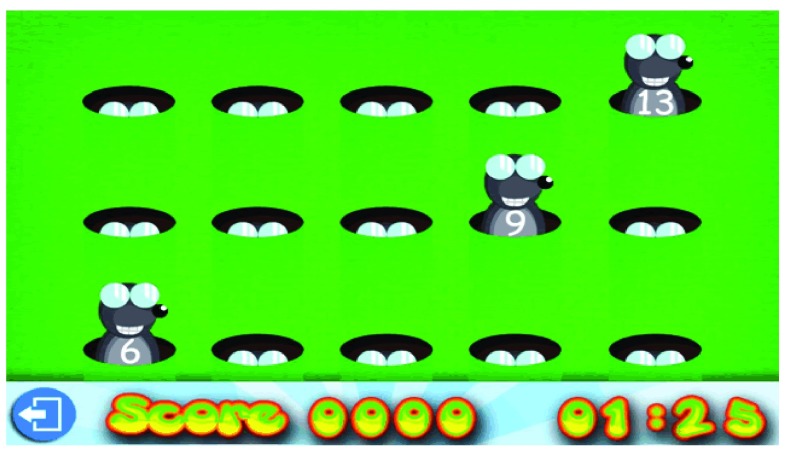
Capture of one of the games based in Trail Making Test.

During the first month of cognitive training therapy, the patient was only allowed to play with specific games based on the TCT Method, using the “ADHD Trainer” ( Figure 2). The patient had to use the app every day at the same time, provided the other targets that were assigned in therapy, such as the progressive reduction in the number of hours to play other games and just being able to play with them once a week, were met. During the first month, he was allowed to play this game to a maximum range of 4 hours per day. No addiction symptoms to this videogame was observed during the first month (tolerance, withdrawal or functional impairment). The average number of hours that the child played the video game was 1 hour a day. In the following months the objective was to play the game at least 10 minutes per day.

In less than two months the video games abuse was substantially reduced, limiting their use to weekends, and always for periods not exceeding 4 hours in total. Although 4 hours a day might is a an important amount of time for a single day, the global reduction of the time wasted in videogames and its limitation to the weekend means a significant improvement in this particular case.

Behavioral and academic improvement was rated on the Conners Parent and Teacher Rating Scales and Barkley School Situations Questionnaire. The initial score of the Conners was 19 for the teachers and 20 for the parents, and after the cognitive training the scores were 15 for the teachers and 16 for the parents. The main severity score for the Barkley School Situations Questionnaire was 70 before starting the training, and after the cognitive training the score was 66.

Both the school and the family reported a significant improvement in the patient after 6 months of TCT cognitive training, which included important improvements of both academic and behavioral outcomes.

## Discussion

Most of the studies reported so far emphasize the potential addictive risk of new technologies and the influence they have on children's interpersonal development, by reducing the time children spend outside home and increasing the time they spend alone playing in front of a television or a computer screen ^12^. It is also known that the new technologies may affect children's academic performance by reducing the number of hours that they dedicate to studying.

There are few studies which focus on the positive aspects of new technologies and the opportunities that they offer new ways of interaction between professionals and users as well as the development of new therapeutic methods, capable of reaching the young.

New technologies, in particular video games, can be used as therapeutic tools to train executive functions ^6, 7^. As they generate greater motivation in children and adolescents they will increase the frequency of performing cognitive tasks oriented to enhance executive functions, especially the working memory. Previous computerized methods have been purposed and have shown to be better than traditional ones ^13, 14^.

There are key advantages for children practicing the TCT Method relative to traditional cognitive training therapies which include:

1) Increased motivation in children for completing cognitive training therapy. This increase in motivation comes from: entertainment value (these games are designed to be similar to regular video games that children enjoy) and feedback on performances relative to own and peer scores (which improves children’s sense of agency and self-efficacy, as demonstrated by documented research on motivation and learning) ^12, 15^.

2) Ease of accessing the application. Children can play the games at any place or time, day and night.

## Conclusion

ADHD patients are especially vulnerable to develop video gaming addition. ADHD patients often suffer from working memory and executive function dysfunctions, but we have observed that very few cognitive training techniques have been developed for ADHD patients in the last years. Poor completion rates of cognitive training in children with ADHD have been observed. We suggest that a daily cognitive computerized training in ADHD patients may improve some of their cognitive symptoms, and might be helpful for treating the video gaming addition.

## Consent

Written informed consent to publish this report was obtained by the patient’s parents.

Dr. Tajima takes responsibility for the integrity of the data and informed consent.
