# Supplementary material for: Case Report: "ADHD Trainer": the mobile application that enhances cognitive skills in ADHD patients
Source: F1000Res. 2015 Jun 23;3:283. Originally published 2014 Nov 18. [Version 3] doi: 10.12688/f1000research.5689.3 (PMC4765719; doi:10.12688/f1000research.5689.3)
Supplement: Peer review history

# Peer review: Referee response for version 3

**DOI:** 10.5256/f1000research.7195.r9143

**Published:** 2015-06-26

**Authors:** Kevin J. Black

**Affiliations:** 1 Department of Psychiatry, Washington University in St. Louis, St Louis, MO, USA

The authors have addressed points #1 and 4 from my previous review, but the following issues remain.

2. The paper still needs copy editing to reflect standard written English. The last 2 sentences of the Introduction provide an example.

- Comma placement:  Do the authors mean that video game play reduces Internet use, or do they mean that MPH is given for both ADHD and video game play?
- "found reduce" -> "found to reduce"
- Here is one potential way to edit the last sentence (insertions marked with bold; deletions marked with underline and subscript).  "Despite the risk of Internet addiction **,** we hypothesize that _good use of _these new technologies can be useful _to develop_ **as **new methods of cognitive training _useful in _to treat ADHD an **d** Internet addiction." 

3a. The exercises comprising "ADHD Trainer" are not described in any detail beyond listing the categories of mental functioning that each task is thought to reflect. For instance, what task is used for "Attention"? Has this implementation of the task been validated elsewhere, or adapted with unpublished modifications from a published task? And so on for each of the categories listed in the Table.

3b. As with any method, the authors need to provide some kind of information about where the reader can obtain "ADHD Trainer".

I have one additional comment that I forgot to add to the previous review.

5. The authors should acknowledge, perhaps in Conclusion, that behavioral interventions other than ADHD Trainer itself may account in part or in whole for the clinical improvement. Other interventions the child received include the following. "The patient was only allowed to play with" ADHD Trainer. "The patient had to use the app every day at the same time." The patient had to meet "the other targets that were assigned in therapy" including a "progressive reduction in the number of hours to play other games" and limiting other game play to once a week.

I have read this submission. I believe that I have an appropriate level of expertise to confirm that it is of an acceptable scientific standard, however I have significant reservations, as outlined above.

# Peer review: Referee response for version 2

**DOI:** 10.5256/f1000research.6850.r8915

**Published:** 2015-06-15

**Authors:** Kevin J. Black

**Affiliations:** 1 Department of Psychiatry, Washington University in St. Louis, St Louis, MO, USA

This is an interesting and encouraging report, but I have the following reservations.

1. One case provides very limited evidence for efficacy and even less for safety. In this light, a couple of statements, including the following, are too enthusiastic and need to be toned down:
  "regular cognitive computerized training in ADHD patients can improve some of their cognitive symptoms and can help treating video game addiction"
  "We conclude that a daily cognitive computerized training in ADHD patients can improve some of their cognitive symptoms, and can help treating the video gaming addiction."
2. ​The manuscript is understandable, but needs copy editing by a native English speaker. For instance, the first sentence of the abstract has 2 errors, and the following phrase is really hard to parse: "the method of Tajima Cognitive Method (TCT) cognitive training called 'ADHD Trainer'."
3. The exercises comprising "ADHD Trainer" are not described in any detail beyond listing the categories of mental functioning that tasks were thought to reflect. If another publication or thesis describes it, a reference would suffice; otherwise a list of tasks would be a first step. Similarly, as with any method, the authors need to provide some kind of information about where the reader can obtain the TCT. 
4. The Barkley School Situations Questionnaire was administered, but the scores are not reported.

I have read this submission. I believe that I have an appropriate level of expertise to confirm that it is of an acceptable scientific standard, however I have significant reservations, as outlined above.

# Response

**Published:** 2015-06-18

**Authors:** Kazuhiro Tajima-Pozo

**Affiliations:** Hospital Fundacion Alcorcon, Spain

According to the reviewer comments

We have included a table showing the cognitive training areas that are trained everyday.

We have made a overall review of the grammar.

We have included the scores of the school situations questionnaire.

Thanks for your review Mr. Black, and best regards.

# Peer review: Referee response for version 2

**DOI:** 10.5256/f1000research.6850.r8583

**Published:** 2015-05-18

**Authors:** Aviv Weinstein

**Affiliations:** 1 Department of Behavioural Sciences, Ariel University, Ariel, Israel

The authors have revised the manuscript to my full satisfaction

I have read this submission. I believe that I have an appropriate level of expertise to confirm that it is of an acceptable scientific standard.

# Response

**Published:** 2015-05-18

**Authors:** Kazuhiro Tajima-Pozo

**Affiliations:** Hospital Fundacion Alcorcon, Spain

Thanks for your comments and your review. Best regards.

# Peer review: Referee response for version 1

**DOI:** 10.5256/f1000research.6082.r6739

**Published:** 2014-11-25

**Authors:** Aviv Weinstein

**Affiliations:** 1 Department of Behavioural Sciences, Ariel University, Ariel, Israel

This is an interesting case report on the usefulness of cognitive computer training for a child who is diagnosed with ADHD and concurrent videogame addiction. The rationale for the study, methods and findings are fine but I would like to make some additional comments.

First, the usefulness of treatment of ADHD and internet addiction by using methylphenidate was reported by Han D *et al., *2009, please add it to the introduction.

Second, what evidence have you got that the child is not addicted to the educational game?

Third, 4 hours of play of a videogame post-treatment is still a lot, this should be mentioned as a limitation.

Fourth,  why were the Conners ratings after treatment for parents and teachers lower compared with pre-treatment?

Fifth, the authors should be commended for the use of advanced computer games for treatment for ADHD. There are other tools for this purpose that are worthwhile mentioning such as ONTRAC ( Mishra *et al.,* 2013) and  the game reported by Prins PJ *et al*., 2011.

I have read this submission. I believe that I have an appropriate level of expertise to confirm that it is of an acceptable scientific standard, however I have significant reservations, as outlined above.

# Response

**Published:** 2014-12-10

**Authors:** Kazuhiro Tajima-Pozo

**Affiliations:** Hospital Fundacion Alcorcon, Spain

We have included the following new references and made the changes according to the reviewer comments.  

Mishra J, Merzenich MM, Sagar R. Accessible online neuroplasticity-targeted training for children with ADHD. Child Adolesc Psychiatry Ment Health. 2013 Nov 14;7(1):38

Prins PJ, Dovis S, Ponsioen A, ten Brink E, van der Oord S. Does computerized working memory training with game elements enhance motivation and training efficacy in children with ADHD? Cyberpsychol Behav Soc Netw. 2011 Mar;14(3):115-22.

Han DH1, Lee YS, Na C, Ahn JY, Chung US, Daniels MA, Haws CA, Renshaw PF.

The effect of methylphenidate on Internet video game play in children with attention-deficit/hyperactivity disorder.

# Response

**Published:** 2015-04-13

**Authors:** Kazuhiro Tajima-Pozo

**Affiliations:** Hospital Fundacion Alcorcon, Spain

We have included the following new references and made the changes according to the reviewer comments.  

Mishra J, Merzenich MM, Sagar R. Accessible online neuroplasticity-targeted training for children with ADHD. Child Adolesc Psychiatry Ment Health. 2013 Nov 14;7(1):38

Prins PJ, Dovis S, Ponsioen A, ten Brink E, van der Oord S. Does computerized working memory training with game elements enhance motivation and training efficacy in children with ADHD? Cyberpsychol Behav Soc Netw. 2011 Mar;14(3):115-22.

Han DH1, Lee YS, Na C, Ahn JY, Chung US, Daniels MA, Haws CA, Renshaw PF.

The effect of methylphenidate on Internet video game play in children with attention-deficit/hyperactivity disorder.
